# Therapy targeted to the metastatic niche is effective in a model of stage IV breast cancer

**DOI:** 10.1038/srep45060

**Published:** 2017-03-21

**Authors:** Byunghee Yoo, Amol Kavishwar, Ping Wang, Alana Ross, Pamela Pantazopoulos, Michael Dudley, Anna Moore, Zdravka Medarova

**Affiliations:** 1 Molecular Imaging Laboratory, MGH/MIT/HMS Athinoula A. Martinos Center for Biomedical Imaging, Massachusetts General Hospital and Harvard Medical School, Boston, MA 02129, USA; 2 TransCode Therapeutics, Inc., Boston, MA 02124, USA

## Abstract

Treatment of stage IV metastatic breast cancer patients is limited to palliative options and represents an unmet clinical need. Here, we demonstrate that pharmacological inhibition of miRNA-10b - a master regulator of metastatic cell viability – leads to elimination of distant metastases in a mouse model of metastatic breast cancer. This was achieved using the miRNA-10b inhibitory nanodrug, MN-anti-miR10b, which consists of magnetic nanoparticles, conjugated to LNA-based miR-10b antagomirs. Intravenous injection of MN-anti-miR10b into mice bearing lung, bone, and brain metastases from breast cancer resulted in selective accumulation of the nanodrug in metastatic tumor cells. Weekly treatments of mice with MN-anti-miR-10b and low-dose doxorubicin resulted in complete regression of pre-existing distant metastases in 65% of the animals and a significant reduction in cancer mortality. These observations were supported by dramatic reduction in proliferation and increase in apoptosis in metastatic sites. On a molecular level, we observed a significant increase in the expression of HOXD10, which is a known target of miRNA-10b. These results represent first steps into the uncharted territory of therapy targeted to the metastatic niche.

Treatment options for patients with metastatic breast cancer are severely limited and ultimately rely on palliative care. Current clinical trials mostly use combinations of different chemotherapeutics targeting various aspects of carcinogenesis, but do not affect the metastatic niche specifically. Studies from several laboratories, including our own, demonstrated that microRNAs play a significant role in the formation of metastasis^1^,^2^. As such, miR-10b has been identified as a key initiator of breast cancer cell migration and invasion^1^,^2^. However, we have recently discovered that in addition to promoting the formation of metastasis, miR-10b is also responsible for viability and proliferation in metastatic cells^1^,^2^. This new role of miR-10b was validated in thorough mechanistic studies showing that inhibition of miRNA-10b led to cell death through apoptosis^1^,^2^. Importantly, this observation was specific to the metastatic niche since inhibition of miRNA-10b in the primary tumor did not affect proliferation or apoptosis^1^,^2^. This led to our hypothesis that therapeutic inhibition of miR-10b would have a profound effect on the growth and viability of established metastatic lesions.

This hypothesis formed a cornerstone of our therapeutic strategy aimed at specific eradication of metastatic tumor cells – cells that have acquired the ability to escape into the circulation, survive during transit, and colonize a distant vital organ, such as the lungs, bone, and brain. These cells express an abnormally high level of miRNA-10b, which allows them to survive in the foreign microenvironment of a distant organ in the process of colonization. In fact, studies showed that miRNA-10b is upregulated in metastatic samples from breast cancer patients compared to matched primary tumors^3^.

As pointed above, recent studies by our group have demonstrated that inhibition of miRNA-10b in metastatic cells leads to their death following upregulation of pro-apoptotic factors^2^. This was achieved using a nanodrug that specifically inhibits microRNA-10b. This nanodrug (termed MN-anti-miR10b) consists of iron oxide nanoparticles (MN) conjugated to LNA-based antagomirs targeting miRNA-10b. In combination with low-dose chemotherapy, MN-anti-miR10b caused persistent regression and complete elimination of lymph node metastases in the MDA-MB-231 breast cancer model with no evidence of systemic toxicity^4^. This model represents early clinical stages of breast cancer, which are associated with a relatively high 5-year survival rate (100% for Stages 0/I, 93% for Stage II and 72% for Stage III). However, the situation for patients with Stage IV metastatic breast cancer is far more dismal with a 5-year survival rate of about 22%. Current treatment regimens for these patients can only extend their life without providing a cure. Ultimately, these patients succumb to spreading metastases and rely on palliative care only. Clearly, there is an unmet clinical need to treat these patients and achieve curable outcomes.

With that in mind the present study investigated whether MN-anti-miR10b could provide a curable outcome in a model of Stage IV metastatic breast cancer. To better mimic a clinical scenario, we used a mouse tumor model, in which murine 4T1 cells were orthotopically implanted into immunocompetent Balb/c animals. This model closely mimics naturally occurring stage IV human breast cancer in terms of colonization of distant organs. By the time the primary tumor is palpable, the tumor cells have metastasized to multiple distant organs, including lymph nodes, blood, liver, lung, brain, and bone. At the same time, unlike human cancer, this model is extremely fast spreading with first metastasis appearing by 2 weeks after primary tumor inoculation. Because of this, the 4T1 orthotopic model growing in an immunocompetent setting is recognized as a most challenging breast tumor model in which to evaluate the efficacy of novel therapies^5^.

Weekly treatments of mice with MN-anti-miR-10b and low-dose doxorubicin resulted in regression of pre-existing distant metastases in 65% of the animals. This translated into a significant reduction in mortality in this treatment group relative to control groups, including a group treated with monotherapy of standard dose doxorubicin, which represents standard-of-care.

We believe that these results represent a substantial step towards finding a cure for Stage IV breast cancer patients and fill a significant gap in the current therapeutic approach by developing a strategy specific to the metastatic tumor cell.

## Methods

### Nanodrug synthesis and characterization

The LNA antagomirs used in this study were designed and synthesized by Exiqon Inc. (Woburn, MA). The 5′-Thiol-Modifier C6 disulfide (5′-ThioMC6) was inserted into both the anti-miR10b and scrambled oligos for conjugation to magnetic nanoparticles. The disulfide on the oligonucleotide was activated by 3% Tris (2-carboxyethyl) phosphine hydrochloride (TCEP, Thermo Scientific Co., Rockford, IL), followed by purification with ammonium acetate/ethanol precipitation treatment prior to conjugation to the nanoparticles, as described previously^2^,^6^.

Aminated magnetic nanoparticles were synthesized following a protocol published previously^1^,^2^. Nanoparticles with a size of 20.3 ± 0.6 nm were used for conjugation to the oligonucleotides. The magnetic nanoparticles were conjugated to the heterobifunctional linker N-succinimidyl 3-[2-pyridyldithio]-propionate (SPDP; Thermo Scientific Co., Rockford, IL) and activated oligos sequentially. Briefly, SPDP was dissolved in anhydrous DMSO and incubated with magnetic nanoparticles. The 5′-ThioMC6 of the oligo was activated to release the thiol via 3% TCEP treatment in nuclease-free PBS. The oligos were purified using an ammoniumacetate/ethanol precipitation method. After TCEP activation and purification, the oligos were dissolved in water and incubated with the SPDP-modified magnetic nanoparticles overnight. The number of oligos per magnetic nanoparticle was determined as 8.0 ± 0.7 using the electrophoresis analysis method described previously^2^,^6^. Both MN-anti-miR10b nanodrug (active nanodrug) and nanodrug containing an irrelevant sequence (MN-scr-miR, inactive nanodrug) were synthesized.

For some experiments the dextran coat of the nanoparticles was labeled with the Cy5.5 near infrared fluorescent (NIRF) dye as previously described^2^.

### Cell culture

Luciferase-expressing 4T1-Red-Fluc breast cancer cell line, derived originally from mouse mammary gland adenocarcinoma, was obtained from Perkin Elmer (Hopkinton, MA). The tumor growth and metastasis of 4T1 cells in BALB/c mice reflect those in humans and are widely used as a model for stage IV human breast cancer. The cells were cultured in high-glucose DMEM supplemented with 5% FBS and antibiotics (100 units/mL penicillin and 100 mg/mL streptomycin), as recommended by the supplier.

To determine the effect of MN-anti-miR10b on breast cancer cells derived from multiple metastatic sites, the following cell lines were used: human MDA-231-BoM-1833, MDA-231-LM2-4175, and MDA-231-BrM2-831 metastatic to bone, lungs, and brain (kindly provided by Dr. Joan Massague, Memorial Sloan Kettering Cancer Center, New York City, NY) and MDA-MB-231-luc-D3H2LN metastatic to the lymph nodes (Perkin Elmer, Hopkinton, MA). All cell lines were cultured in DMEM supplemented with 10% FBS and 1% Penicillin-Streptomycin. Cells were added to 8 –well chambered slides (Millicell EZ, Millipore, Billerica, MA). Nanodrugs were added in duplicates such that the final concentration of nanodrug was 800, 400, 200, and 100 nM. Wells treated with inactive nanodrug (MN-scr-miR), parental MN nanoparticles, and untreated wells were used as controls. Slides were incubated at 37 °C in a CO_2_ incubator. After 48 hrs, the cells were fixed with 4% formaldehyde, stained with DAPI and observed under a fluorescence microscope. Three fields of view were randomly selected for each well and analyzed by ImageJ (Ver. 1.48, Bethesda, MD).

### Animal model

Eight-week-old female Balb/c mice (The Jackson Laboratory; Bar Harbor, ME) were implanted orthotopically under the top left third mammary fat pad with 4T1-Red-Fluc cells (0.5 × 10^6^ cells). In this model, tumor cells can spontaneously metastasize from the primary tumor in the mammary gland to multiple distant sites including lymph nodes, blood, liver, lung, brain, and bone. Noninvasive bioluminescence imaging (BLI) was used for monitoring tumor growth and appearance of metastases (described below). Akin to clinical treatment of metastatic breast cancer, the primary tumors were surgically removed once distant metastases were confirmed by BLI. Therapy started on the day of primary tumor removal and was repeated once a week as described below. All animal experiments were performed in compliance with institutional guidelines and approved by the Institutional Animal Care and Use Committee at Massachusetts General Hospital (Boston, MA).

### Near Infrared Fluorescence Optical Imaging (NIRF)

Imaging was performed using the IVIS Spectrum imaging system (Perkin Elmer, Hopkinton, MA). Anesthetized mice were injected i.v. with Cy5.5 labeled nanodrug (30 mg/kg as iron, 20 mg/kg as oligo) and scanned by fluorescence reflectance imaging 24 hrs later. Organs were harvested and imaged *ex vivo*. The acquisition conditions are summarized as follows: Exposure time, 0.5 sec; Binning factor, 8; Excitation filter range, 675 nm; Emission filter range, 720 nm; f number, 2. All images were analyzed using the Living Image Software (ver 4.5, IVIS Spectrum, Perkin Elmer, Hopkinton, MA). Radiant efficiency of the excised tissues was used for signal quantification. At the end of the fluorescence imaging session, mice were injected intraperitoneally with D-Luciferin potassium salt and subjected to bioluminescence imaging.

### Bioluminescence optical imaging (BLI)

BLI was used to evaluate metastatic burden. Imaging was performed using the IVIS Spectrum imaging system. Anesthetized mice were injected intraperitoneally with D-luciferin potassium salt in DPBS (200 mL of 15 mg/mL; Perkin Elmer, Hopkinton, MA) 12 minutes before image acquisition. Identical imaging acquisition settings (time, ~ 0.5–60 seconds; F-stop, 2; binning, medium) and the same ROI were used to obtain total radiance (photons/sec/cm^2^/sr) over the whole body. BLI was performed for about 6 to 12 minutes to obtain the maximum radiance. All images were processed using the Living Image Software (ver 4.5, IVIS Spectrum, Perkin Elmer, Hopkinton, MA). The total radiance from the bioluminescence readings was used for signal quantification.

### Imaging of Nanodrug Accumulation in Distant Organs

Studies were performed in immunocompetent Balb/C mice implanted orthotopically with luciferase-expressing 4T1-Red-Fluc cells. To monitor delivery, the nanodrug was labeled with the near-infrared (NIRF) fluorescent dye, Cy5.5, which was loaded onto the nanodrug as described in *Nanodrug Synthesis and Characterization*. The nanodrug was injected intravenously. Twenty four hours after injection, the animals were sacrificed, organs were excised and imaged by fluorescence reflectance imaging as described above. Imaging involved bioluminescence optical imaging (to localize tumor cells) and NIRF optical imaging (to localize the nanodrug).

### Therapy

Therapy was delivered to animals immediately after the surgical removal of primary tumors, once distant metastases were confirmed by BLI. The therapeutic protocol consisted of concurrent injections of MN-anti–miR-10b or MN-scr-miR intravenously (30 mg/kg as iron, 20 mg/kg as oligo), and doxorubicin (intraperitoneally; 2 mg/kg for low-dose or 8 mg/kg for high dose; Sigma, St Louis, MO). The following treatment groups were used: Group 1: PBS only (n = 3), Group 2: High-dose doxorubicin (n = 6), Group 3: MN-scr-miR with low-dose doxorubicin (n = 13), and Group 4: MN-anti–miR-10b with low-dose doxorubicin (n = 17). Therapy was administered weekly until the disappearance of BLI-visible metastasis and was stopped after background BLI radiance had been observed for two consecutive weeks. All mice were monitored weekly by BLI to assess metastatic burden for a maximum of 8 weeks after the first treatment or until animals became moribund.

### Histology and fluorescence microscopy of tissue sections

To analyze the metastatic lesions *post mortem*, excised tissues were embedded in Tissue-Tek OCT compound (Sakura Finetek, Torrance, CA), snap-frozen in liquid nitrogen and cut into 7-μm sections.

To determine MN-anti–miR-10b accumulation in tissue, the sections were stained using incubation with an anti-firefly luciferase antibody (1:50 dilution; Abcam, Cambridge, MA) at 4 °C overnight, followed by incubation with a Texas Red–conjugated goat anti-rabbit secondary antibody (1:50 dilution, Santa Cruz Biotechnology, Santa Cruz, CA) at room temperature for 1 hour. Afterward, the slides were counterstained and mounted with Vectashield mounting medium with DAPI (Vector Laboratories, Inc., Burlingame, CA). The same sections were co-stained with a FITC-conjugated anti-dextran monoclonal antibody (1:50 dilution; Stemcell Technologies, Vancouver, BC, Canada).

Apoptosis in excised tissues was evaluated by performing a terminal deoxynucleotidyl transferase–mediated dUTP nick end labeling (TUNEL) assay (ApopTag Fluorescein *In situ* Apoptosis Detection kit, Chemicon International, Temecula, CA) according to the manufacturer’s protocol.

For determining proliferation, frozen sections were incubated with an anti-Ki67 antibody (1:100 dilution; Abcam, Cambridge, MA) followed by a DyLight-488–labeled secondary antibody at room temperature for 1 hour. Afterward, the slides were counterstained and mounted with Vectashield mounting medium with DAPI.

For monitoring E-cadherin, frozen sections were incubated with an anti-E-Cadherin antibody (1:100 dilution; Abcam, Cambridge, MA) followed by a FITC–labeled secondary antibody at room temperature for 1 hour.

For monitoring HOXD10 expression, frozen sections were incubated with an anti-HOXD10 antibody (1:100 dilution; Abcam, Cambridge, MA) followed by a FITC–labeled secondary antibody at room temperature for 1 hour.

For the other targets including RHOC, PTEN, and TXB5, frozen sections were incubated with an anti-RHOC antibody (1:100 dilution; Abcam, Cambridge, MA), an anti-PTEN (1:100 dilution; Santa Cruz Biotechnology, Dallas, TX), and an anti-TXB5 antibody (1:100 dilution; Abcam, Cambridge, MA), respectively, followed by a DyLight 488–labeled secondary antibody at room temperature for 1 hour. Afterward, the slides were counterstained and mounted with Vectashield mounting medium with DAPI.

For histopathology, the tissues were stained with hematoxylin and eosin (H&E). The sections were analyzed by fluorescence microscopy using a Nikon Eclipse 50i fluorescence microscope (Nikon, Tokyo, Japan), equipped with the necessary filter sets (Chroma Technology Corporation, Bellows Falls, VT). Images were acquired using a charge-coupled device camera with NIRF sensitivity (SPOT 7.4 Slider RTKE; Diagnostic Instruments, Sterling Heights, MI). The images were analyzed using SPOT 4.0 Advance version software (Diagnostic Instruments, Sterling Heights, MI).

### Statistical analysis

Data were expressed as mean ± SD or SEM, where indicated. Statistical comparisons were drawn using a two-tailed t test or ANOVA, where indicated. A value of P < 0.05 was considered statistically significant.

## Results

### Nanodrug Delivery to Distant Metastases

**Figure 1:**
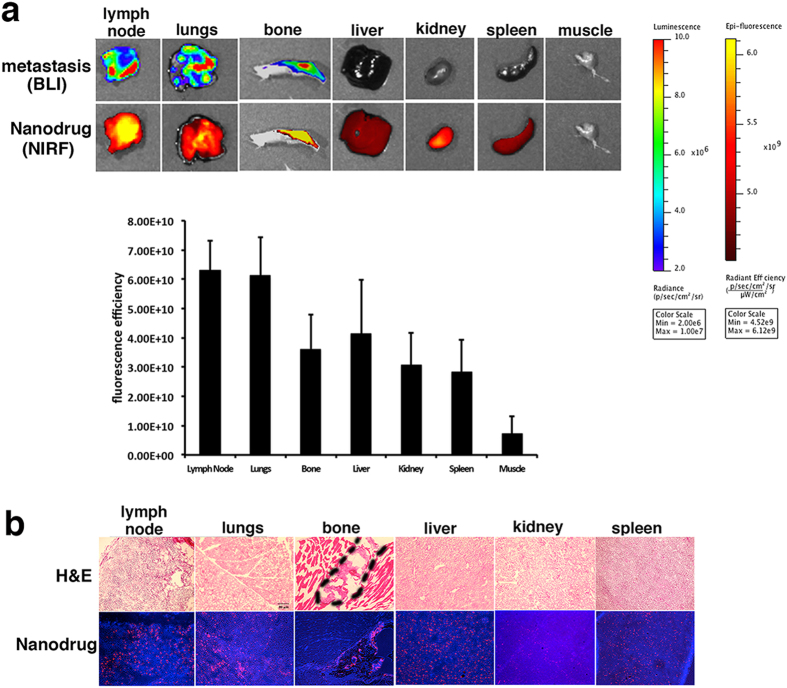
Nanodrug accumulation in metastatic organs. (**a**) Top: Macroscopic imaging of metastatic burden (bioluminescence imaging, BLI) and nanodrug uptake (fluorescence reflectance imaging, Fl) in excised organs. Bottom: Quantitative analysis of fluorescence intensity in major organs). Metastatic organs were associated with high uptake of the nanodrug. Additional organs of high uptake reflected the natural pathway of metabolism and excretion of the nanoparticles. (**b**) Histology of tissue sections derived from excised organs. Top: H&E, bottom: fluorescence (Nanodrug: red, Cy5.5; nuclei: blue, DAPI). For lymph nodes and lungs, the whole tissue shown in the image is a metastatic lesion. The bone metastatic lesion is outlined in the image.

As a first step towards developing a therapeutic approach for stage IV breast cancer, we needed to establish delivery of the MN-anti-miR10b nanodrug to distant metastatic sites. As seen in Fig. 1a, the nanodrug accumulated in distant metastatic organs. As expected, the foci of nanodrug accumulation (Cy5.5 fluorescence) co-localized with the metastatic lesions (bioluminescence, BLI).

Organs that were free of metastases but accumulated the nanodrug included the liver, spleen, and kidney. This result is not unexpected. The nanoparticles that form the core of the nanodrug are taken up by cells of the reticuloendothelial system, found in the liver and spleen, and rapidly broken down^7^. The iron from the core of the nanodrugs enters the endogenous iron pool, whereas the dextran from the nanodrug coating is cleared through the kidneys. Finally, muscle tissue commonly used as a reference in this type of study, did not show evidence of nanodrug accumulation, as anticipated based on the fact that it has a relatively hypopermeable vasculature (Fig. 1a).

Fluorescence microscopy of the same organs confirmed the high level of accumulation of the nanodrug in the metastatic lesions. It also corroborated the expected uptake by cells in the liver, spleen, and kidney (Fig. 1b).

In this initial cohort of mice, no brain metastases were observed within the timeline of the experiment. However, in the later therapeutic studies, which were longer-term, some of the mice formed brain metastases. Uptake of the nanodrug by brain micrometastases was assessed by immunofluorescence. Accumulation of the nandrug was pronounced in brain micrometastases similarly to other distant metastatic sites (Suppl. Fig. 1). These studies provided further proof of nanodrug entry into metastatic cells, which is a prerequisite for its effectiveness.

### Therapeutic Effect of the Combination Treatment with MN-anti-miR10b and Low-dose Doxorubicin

To investigate whether MN-anti-miR10b has the potential to provide a curable outcome to Stage IV breast cancer patients, we performed studies in immunocompetent mice with established metastases. Similar to the clinical treatment of metastatic breast cancer, the primary tumors were surgically removed in these mice once metastases were confirmed by bioluminescence imaging.

**Figure 2:**
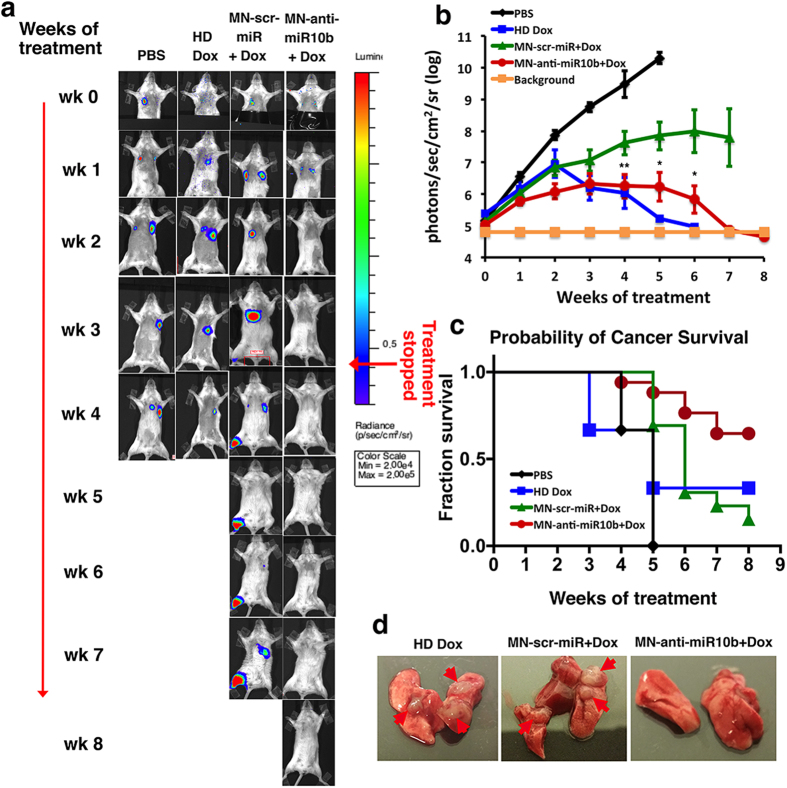
Metastatic burden and survival of mice treated with MN-anti-miR10b and low-dose doxorubicin. (**a**) Representative bioluminescence images of metastatic burden showing evidence of complete regression of metastases in animals treated with MN-anti-miR10b and low-dose doxorubicin. The images shown are representative of the results observed in the majority of animals in each group. (**b**) Quantitative analysis of relative metastatic burden (photon flux over the whole body) from all treatment groups indicating complete regression of metastases in experimental animals treated with MN-anti-miR10b and low-dose doxorubicin. (Data represent average ± s.e.m.; Within-Subjects ANOVA: *p < 0.05; **p < 0.001; values were compared to MN-scr-miR and low-dose doxorubicin) (**c**) Mortality. Cancer survival was significantly higher in the animals treated with MN-anti-miR10b and low-dose doxorubicin, compared to control groups. (**d**) Macroscopic images post-necropsy (lungs are shown). The majority of the animals treated with MN-anti-miR10b and low-dose doxorubicin presented with no evidence of lung macrometastases at necropsy. Results represent a summary of three independent trials.

Figure 2 presents the results of treatment. In control mice treated with PBS, metastases progressed rapidly (Fig. 2a and b and Suppl. Fig. 2). None of the animals showed evidence of metastatic regression, defined as reduction in BLI signal to background level (Fig. 2b and Suppl. Fig. 2). This resulted in 100% animal mortality by week 5 (Fig. 2c). In mice treated with inactive nanodrug (MN-scr-miR) in combination with low-dose doxorubicin, metastases grew slower than in the PBS controls (Fig. 2a and b and Suppl. Fig. 2). There was 80% cancer mortality in this group by week 7 (Fig. 2c). By contrast, in the mice treated with the active nanodrug (MN-anti-miR10b) and low-dose doxorubicin, regression of distant metastases was evident by week 6 (Fig. 2a and b and Suppl. Fig. 2). Regression was accomplished in 65% of the animals, whereas the remainder of the animals (35%) progressed despite the fact that metastatic burden at the beginning of treatment was not different between the animals (Fig. 2b and Suppl. Fig. 2). Importantly, the animals that regressed remained metastases free for the course of the experiment even though treatment was stopped after BLI signal over the whole body reached background level.

In animals treated with high-dose doxorubicin, the response was variable. A subset of the animals (33%) achieved metastatic regression (Fig 2a and b and Suppl. Fig. 2). However, this was accompanied by significant morbidity and mortality. Overall, in the group that received high dose doxorubicin, 67% of the animals succumbed by week 5 (Fig. 2c).

Macroscopic observation of the organs post-necropsy in all groups confirmed the observed results. No metastatic lesions could be detected in the responders treated with the active nanodrug in combination with low-dose doxorubicin (Fig. 2d).

Supplemental Fig. 3 shows representative images of individual mice between 4–6 weeks of treatment obtained from one of the three independent trials, summarized in Fig. 2. The figure illustrates the uniformly positive response to treatment with the active nanodrug in combination with low-dose doxorubicin. Specifically, at week 6, four of the mice had no detectable metastasis and one had a small lesion (14.5-fold smaller than the peak metastatic burden observed at week 2). In the group treated with a high dose of doxorubicin, one of the mice in this trial succumbed to metastatic disease as early as week 4 of treatment. A second mouse was alive at week 6 but harbored metastasis. Only one of the mice treated with high-dose doxorubicin had evidence of regression below the limit of detection by bioluminescence imaging or macroscopic observation post-necropsy. Finally, in the control groups treated with PBS or inactive nanodrug in combination with low-dose doxorubicin, metastatic disease progressed uniformly (Suppl. Fig. 2).

An additional observation that stemmed from these studies was that in the mice treated with MN-anti-miR10b and low-dose doxorubicin, there was a significantly lower incidence of detectable multiple organ metastases at the end-point of the study. In the PBS and high-dose doxorubicin groups, the animals died before multiple-organ metastases could be detected. In the animals treated with the inactive nanodrug, MN-scr-miR, and low-dose doxorubicin, which lived beyond 6 weeks, there was evidence of multiple organ metastases at the end-point of treatment. By contrast, in the group treated with MN-anti-miR10b in combination with low-dose doxorubicin, only one animal out of 17, presented with lung metastases and a single bone metastatic lesion (Suppl. Fig. 4). This observation indicated that treatment with the active nanodrug and low-dose doxorubicin had a global effect on metastases at multiple organ sites. The absence of recurrence, even after treatment was discontinued, indicates complete regression of these metastatic lesions.

**Figure 3:**
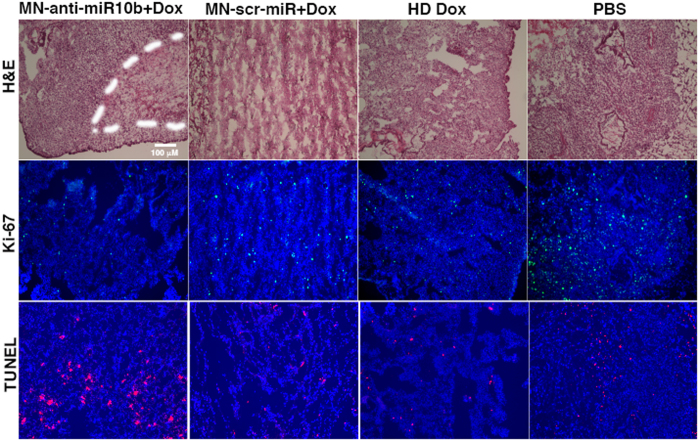
Histological analysis of tumor cell proliferation (Ki-67) and apoptosis (TUNEL) in lung sections from experimental and control animals. There was a visibly higher frequency of Ki-67-negative and TUNEL-positive cells from animals treated with MN-anti-miR10b and low-dose doxorubicin compared to control groups. (DAPI: nuclei, blue; Ki-67: proliferation, green; TUNEL: apoptosis nuclei, red).

*Post mortem* examination focused on the metastatic lungs, since this is the organ most commonly presenting with metastatic lesions in the 4T1 model. Histological analysis confirmed the significantly lower metastatic burden in the experimental mice and revealed both induction of apoptosis and reduction of proliferation in the animals treated with MN-anti–miR10b in combination with low-dose doxorubicin, compared to the control treatments (Fig. 3 and Suppl. Fig. 5). This result is consistent with our earlier findings that miR-10b inhibition causes a dramatic reduction in proliferation and increase in apoptosis in metastatic breast cancer cells^4^.

**Figure 4:**
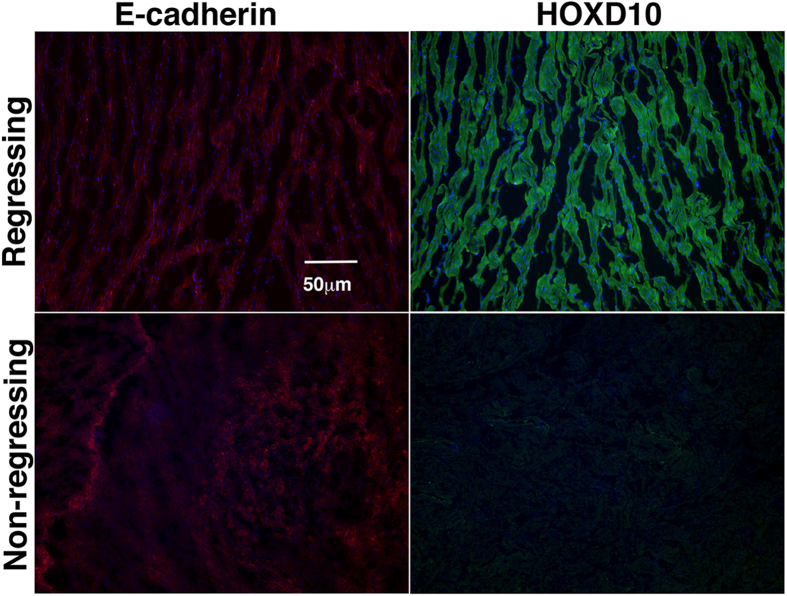
Immunostaining showing the expression of E-cadherin and HOXD10 in lung sections from animals with evidence of metastatic regression and animals that failed to regress lung metastases in response to treatment with MN-anti-miR10b and low-dose doxorubicin. E-cadherin, was highly expressed in the lung metastases of both the regressing and non-regressing groups. The direct miR-10b target, HOXD10, was upregulated in the regressing group compared to the non-regressing group, indicating inefficient inhibition of miR-10b in the animals that failed to regress metastases.

While the combination treatment with MN-anti-miR10b and low-dose doxorubicin resulted in metastatic regression in 65% of the animals, there was a 35% failure in that group to regress established lung metastases. In an attempt to investigate the reasons behind this failure, we analyzed lung metastatic tissues from the animals in this group that did not respond to treatment. In parallel, we looked at tissues from mice with evidence of regression that were sacrificed prior to the complete eradication of detectable metastases. Our analysis involved assessment of E-cadherin, and HOXD10. HOXD10 was selected because it is the most well-known direct target of miR-10b^1^. Its expression is the only direct method to evaluate successful delivery of MN-anti-miR10b, since inhibition of miR-10b by the therapeutic is achieved through the formation of stable heteroduplexes between the antagomir and miR-10b, hence, the need to use HOXD10 expression as a marker for inhibition of miR-10b. By contrast, E-cadherin does not appear to be directly influenced by miR-10b expression, consistent with the literature^8^. Whereas E-cadherin expression appeared similar between regressing and non-regressing animals, the expression level of HOXD10 was markedly higher in the regressing animals relative to the animals that failed to regress (Fig. 4 and Suppl. Fig. 6). This strongly suggested that the failure of some of the animals to regress metastases stemmed from inadequate inhibition of miR-10b by the nanodrug. It also proved that in the responding animals, miR-10b was successfully inhibited.

To further look into the mechanism behind the observed effect mediated by miR-10b inhibition, we analyzed the abundance of additional validated miR-10b targets. These included RHOC^1^, PTEN, and TBX5^9^. Consistent with the literature, we observed a strong inhibition of the pro-metastatic RHOC in the lungs of animals treated with MN-anti-miR10b and doxorubicin, compared to the control groups (Suppl. Fig. 7). The link between miR-10b expression and breast cancer cell proliferation is partially attributed to inhibition of the transcription factor TBX5, leading to repression of the tumor suppressor PTEN^9^. However, our studies failed to provide clear evidence of TBX5 or PTEN upregulation by inhibition of miR-10b, suggesting that an alternative pathway may be behind the observed therapeutic effect in this model (Suppl. Fig. 7).

Finally, as a step towards clinical translation of these findings, we performed preliminary *in vitro* studies in human MDA-MB-231-derived cell lines with tropism to lymph nodes, bone, lungs, or brain (MDA-MB-231-luc-D3H2LN, MDA-231-BoM-1833, MDA-231-LM2-4175, and MDA-231-BrM2-831). As seen in Suppl. Fig. 8, all cell lines were associated with nanomolar IC50 values and were highly sensitive to the pro-apoptotic effect of MN-anti-miR10b, suggesting that miR-10b inhibition has a global effect on the viability of human metastatic tumor cells.

## Discussion

Despite the recent success of targeted therapy and immunotherapy, the cases of lasting regression of stage IV metastatic cancer remain a rare exception. Our approach differs from others in that it targets the metastatic niche.

The specific targeting of the metastatic niche is a function of the therapeutic target, miR-10b. In our earlier studies, we found that miRNA-10b is uniquely linked to tumor cell survival in metastatic cells and in fact represents a master switch of metastatic cell viability^2^,^4^. These prior studies generated thorough mechanistic insight into the precise molecular mechanism behind the promise of miR-10b targeted therapy for metastastic disease. In the present investigation we take advantage of this knowledge and apply the miR-10b-inhibitory nanodrug, MN-anti-miR10b, in a very aggressive model of stage IV metastatic breast cancer (4T1 breast adenocarcinoma). Treatment with the nanodrug in combination with low dose doxorubicin resulted in regression of metastases in 65% of animals.

Previous imaging studies that investigated the cellular kinetics of magnetic nanoparticles demonstrated that they are lost from the tumor cells primarily through dilution as a result of cell division^10^. Since nanoparticles are used as oligo carriers in our approach, this loss would reduce the effective concentration of the nanodrug in cells with a very fast rate of metastatic cell division. To slow down the cell cycle and inhibit nanodrug dilution in the tumor cells, we used low-dose doxorubicin, which is also a clinical standard-of-care for breast cancer. However, in humans, the application of low-dose chemotherapy may not be necessary because of the slower rate of metastatic growth.

Another reason for the anticipated success of this therapy in clinic is based on the fact that even though therapy was discontinued after evidence of complete regression, the animals remained metastases-free for the course of the experiment. Consequently, if we could translate this regimen into the clinic, it would be highly advantageous for patients with impaired liver and kidney function who are more prone to develop complications stemming from potential side effects.

As mentioned above, we believe that the significance of this work rests on its potentially high clinical impact. Through the combination of an effective delivery system and a robust therapeutic target, we have achieved progress in a model of stage IV breast cancer. These findings are important because delivery of oligonucleotide therapy to distant metastatic sites has so far represented an insurmountable challenge. Here we show that the described nanoparticle delivery system and antagomir design can overcome this challenge. The robustness of the delivery system is partly responsible for the success of our therapeutic approach, as compared to earlier attempts based on cholesterol-conjugated 2′-*O*-Me-modified antagomirs^11^.

Secondly, the significance of the work extends from the therapeutic target, which plays a unique role as an essential driver of metastatic cell survival. Combined with our earlier mechanistic studies^2^,^4^, the present findings strongly suggest that, if successfully translated in humans, the described approach could lead to regression of metastatic breast cancer. Additionally, since tumor cells take up the nanodrug independently of their receptor status, this approach could be especially beneficial for patients with triple negative breast cancer for whom treatment options are scarce.

An added value of our approach derives from the fact that the magnetic nanoparticles used as a carrier for the therapeutic oligos, have been used in clinical practice as a contrast agent for magnetic resonance imaging (MRI) since the early 1980 s. Therefore, this property of the nanodrug allows for the monitoring of its delivery, which could be crucial for patients whose therapy is failing for unknown reasons.

Finally, since miRNA-10b plays a proven role in other human metastatic cancers beyond breast cancer^12^,^13^,^14^ including lung, colorectal, gastric, bladder, pancreatic, ovarian, hepatocellular and brain cancer, we believe that our approach is fundamentally focused on the unique biology of the metastatic tumor cell and, therefore, is broadly relevant to metastatic disease beyond breast cancer.

## Additional Information

**How to cite this article**: Yoo, B. *et al*. Therapy targeted to the metastatic niche is effective in a model of stage IV breast cancer. *Sci. Rep.* **7**, 45060; doi: 10.1038/srep45060 (2017).

**Publisher's note:** Springer Nature remains neutral with regard to jurisdictional claims in published maps and institutional affiliations.

## Supplementary Material

Supplementary Dataset 1
