# Supplementary material for: Therapy targeted to the metastatic niche is effective in a model of stage IV breast cancer
Source: Sci Rep. 2017 Mar 21;7:45060. doi: 10.1038/srep45060 (PMC5359550; doi:10.1038/srep45060)
Supplement: Supplementary Dataset 1 [file srep45060-s1.docx]

**Therapy targeted to the metastatic niche is effective in a model of stage IV breast cancer**

Byunghee Yoo^1^, Amol Kavishwar^1^, Ping Wang^1^, Alana Ross^1^, Pamela Pantazopoulos^1^, Michael Dudley^2^, Anna Moore^1*^ and Zdravka Medarova^1*^

^1^*Molecular Imaging Laboratory, MGH/MIT/HMS Athinoula A. Martinos Center for Biomedical Imaging, Massachusetts General Hospital and Harvard Medical School, Boston, MA 02129, USA.*

*^2^TransCode Therapeutics, Inc., Boston, MA 02124, USA.*

* Corresponding author

Zdravka Medarova, Ph.D., Associate Professor of Radiology

Anna Moore, Ph.D., Professor of Radiology

Molecular Imaging Laboratory

MGH/MIT/HMS Athinoula A. Martinos Center for Biomedical Imaging

Department of Radiology

Massachusetts General Hospital and Harvard Medical School, Boston, MA 02129, USA ; Tel: 617-643-4889. Fax: 617-643-4865.

E-mail: [zmedarova@partners.org](mailto:zmedarova@partners.org) or amoore@helix.mgh.harvard.edu

Conflict of Interest Statement: Anna Moore and Zdravka Medarova are Founders, Directors, and Scientific Advisory Board Members of TransCode Therapeutics, Inc. Michael Dudley is CEO and Founder of TransCode Therapeutics, Inc.

Running title:

Prolonged survival after combination therapy for advanced metastatic breast cancer


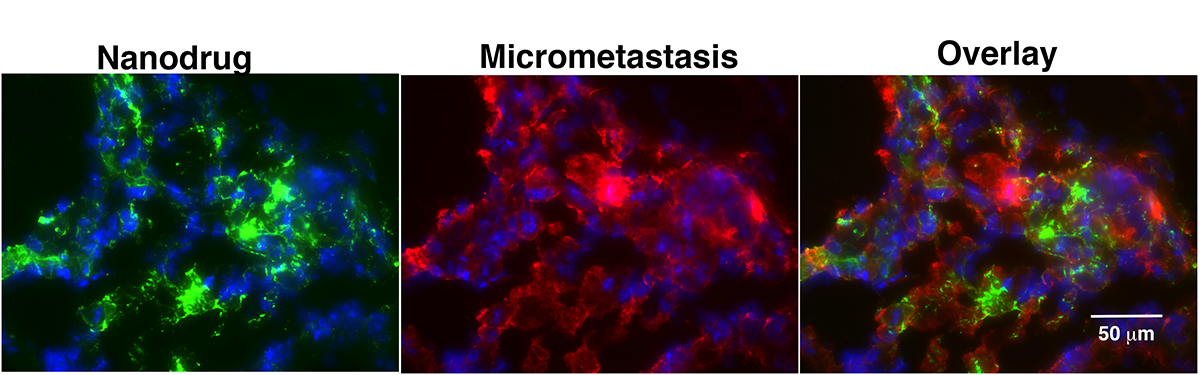


**Supplemental Figure 1.** Immunofluorescence of nanodrug accumulation in brain micrometastases. There was co-localization between the nanodrug (green, dextran) and tumor cells (red, luciferase) in brain micrometastases. Tissues were derived from animals treated with the inactive nanodrug, MN-scr-miR.


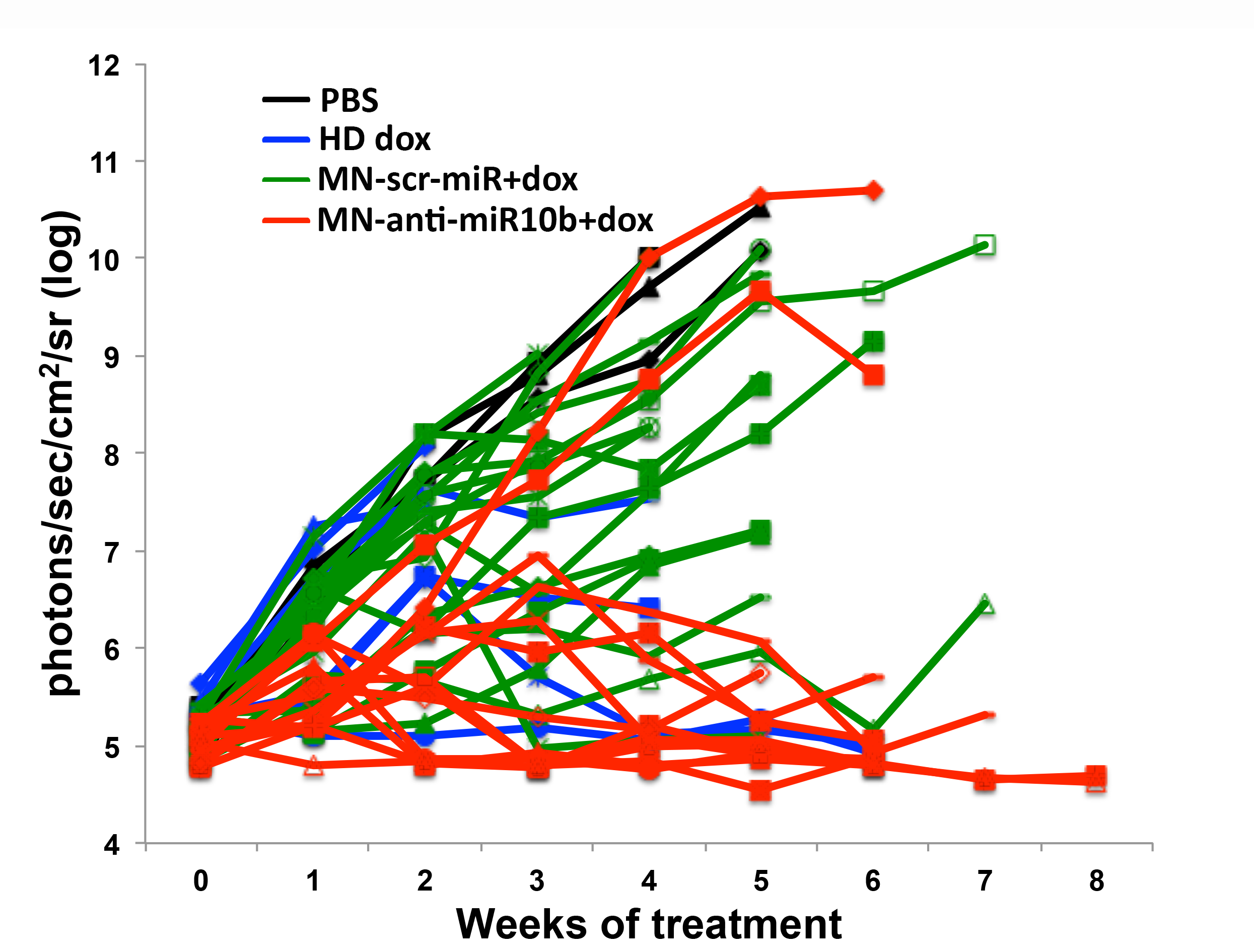


**Supplemental Figure 2.** Quantitative analysis of relative metastatic burden (photon flux over the whole body) from individual mice in the study.


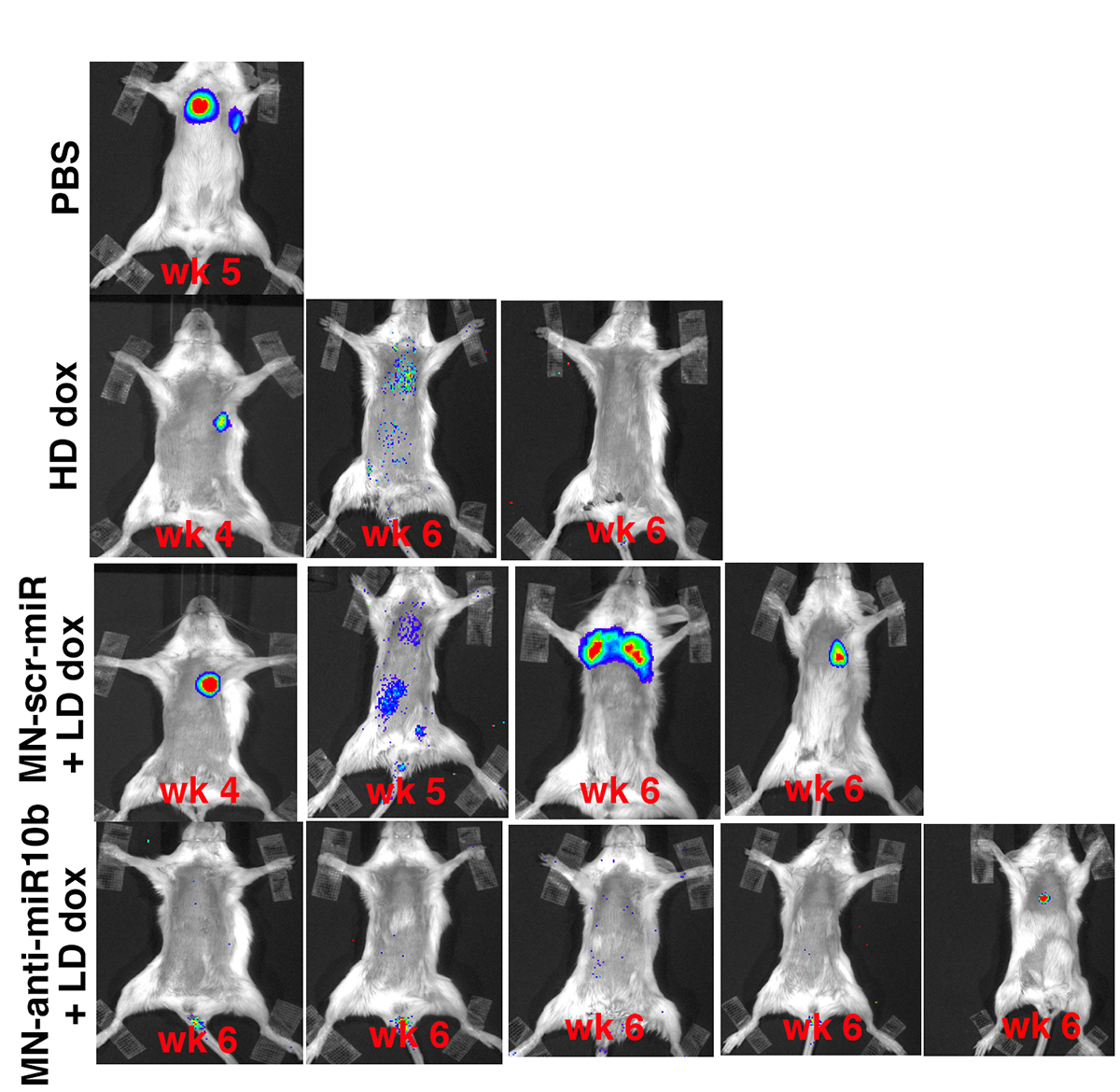


**Supplemental Figure 3.** Metastatic burden in individual mice at the end-point of treatment (trial 1). There was complete regression of metastases in all but one of the mice treated with MN-anti-miR-10b and low-dose doxorubicin. In the control groups treated with PBS or MN-scr-miR and low dose doxorubicin, there was uniform metastatic progression. In the group treated with a high dose of doxorubicin, the response was heterogeneous.


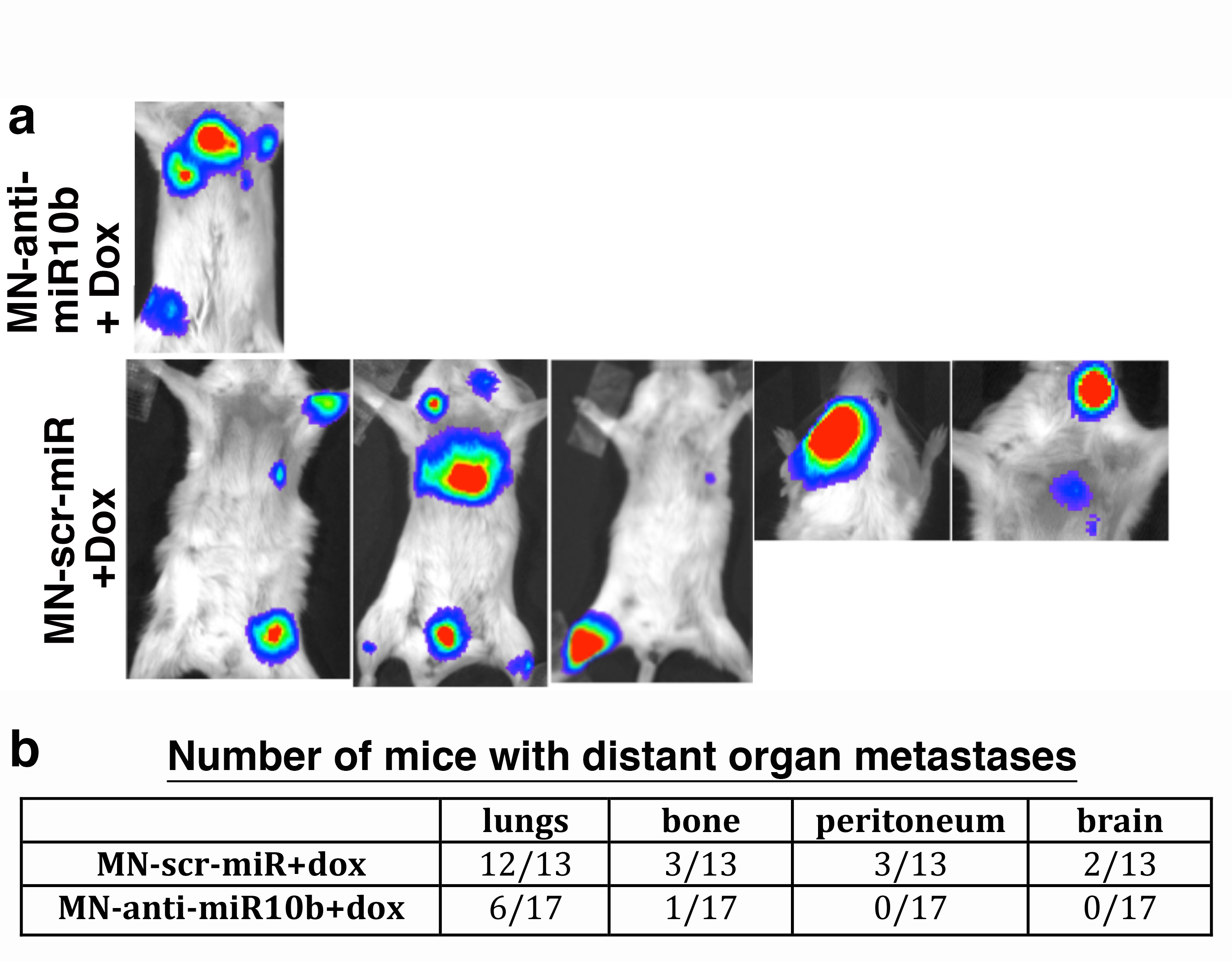


**Supplemental Figure 4.** Evidence of multiple organ metastases in experimental and control animals. **a.** Whole-body BLI images. **b.** Number of animals in each group showing evidence of metastases to the lungs, bone, peritoneum, and brain. Only one of 17 animals treated with MN-anti-miR10b and low-dose doxorubicin showed evidence of multiple-organ metastases at the end-point of treatment.


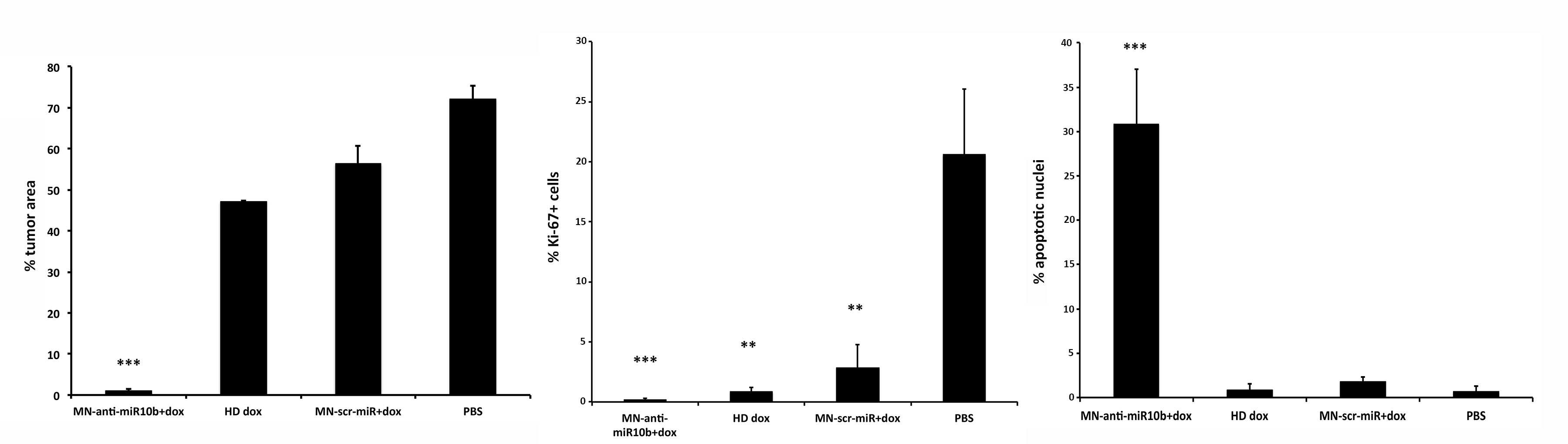


**Supplemental Figure 5.** Quantitative analysis of metastatic burden (from histopathology, left) and relative rates of proliferation (middle) and apoptosis (right) in the study groups. Metastatic burden is shown as % of tissue section occupied by tumor tissue vs total tissue. Proliferation and apoptosis are shown as % of Ki-67/TUNEL-positive cells (Student’s t-test, **, p <0.05, ***, p<0.01, n = 5).


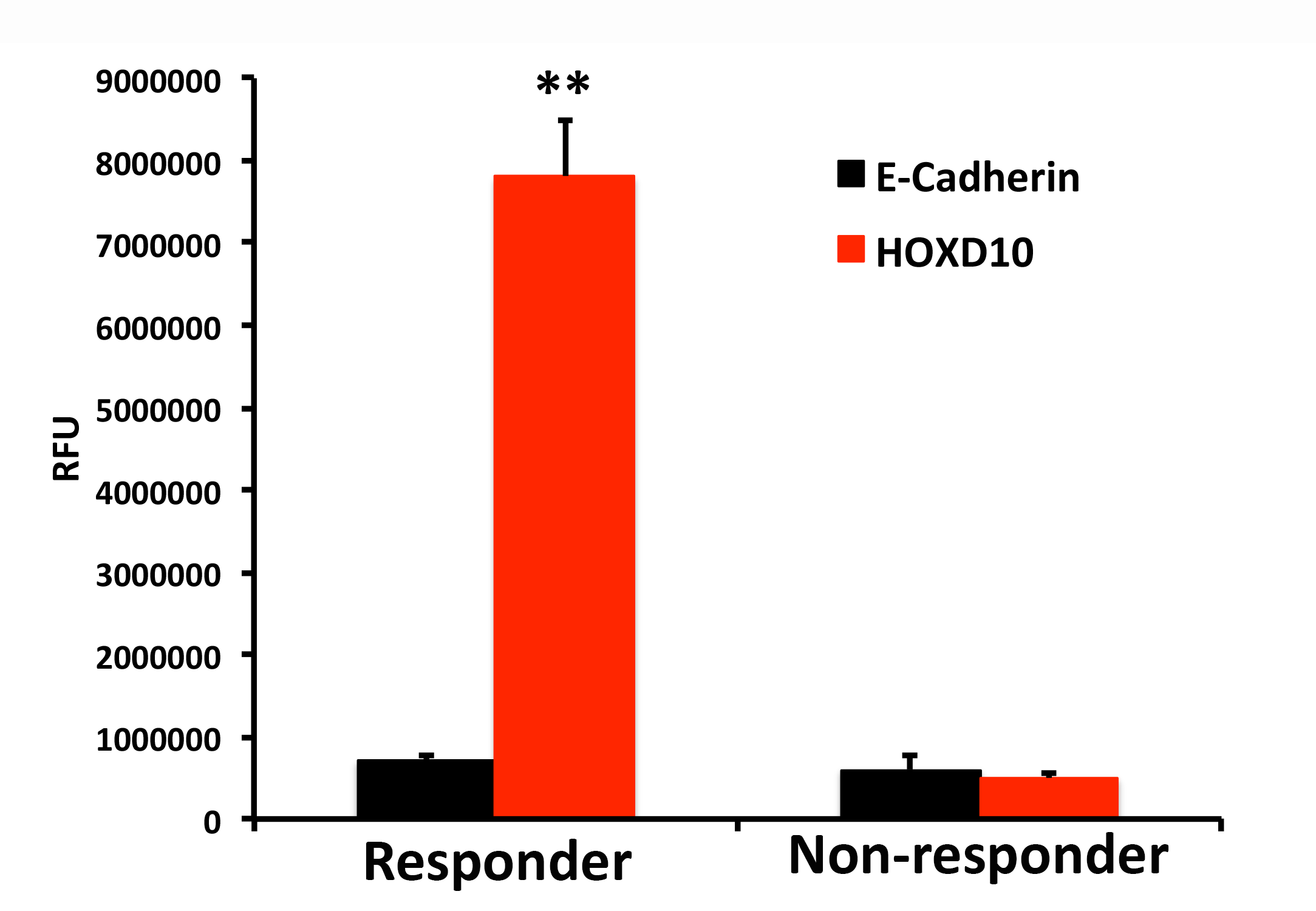


**Supplemental Figure 6.** Quantitative analysis of relative E-cadherin and HOXD10 abundance in histological sections from the study groups. Results represent relative fluorescence intensity in the red (E-cadherin) and green (HOXD10) channels (Student’s t-test, **, p <0.05, n = 5).


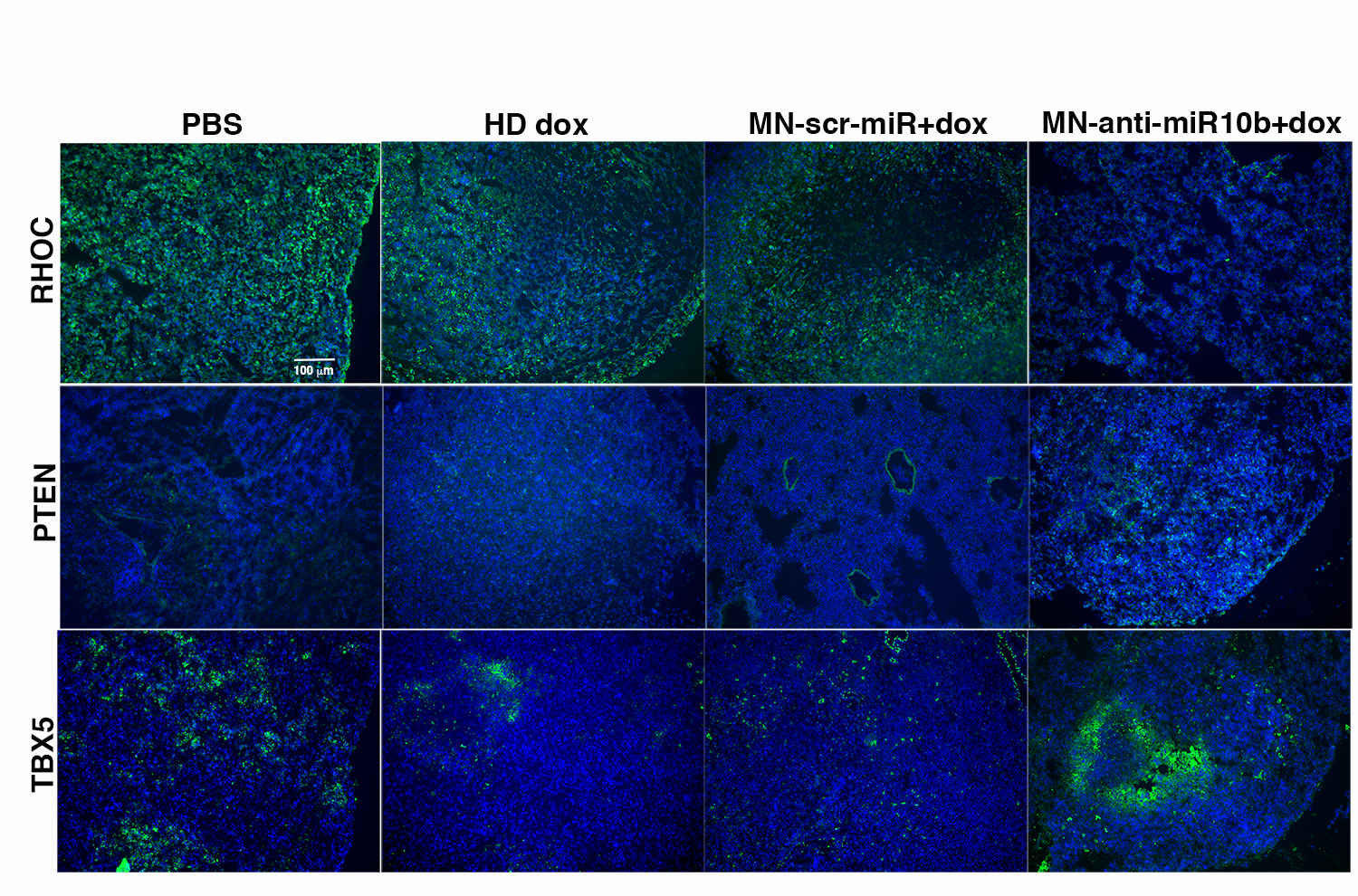


**Supplemental Figure 7.** Immunofluorescence of RHOC, PTEN, and TBX5 in lung sections of mice treated with MN-anti-miR10b+dox. There was clear inhibition of the pro-metastatic RHOC and a moderate induction of PTEN and TBX5, compared to control treatments (green – RHOC, TPEN, TBX5; blue – DAPI, nuclei).


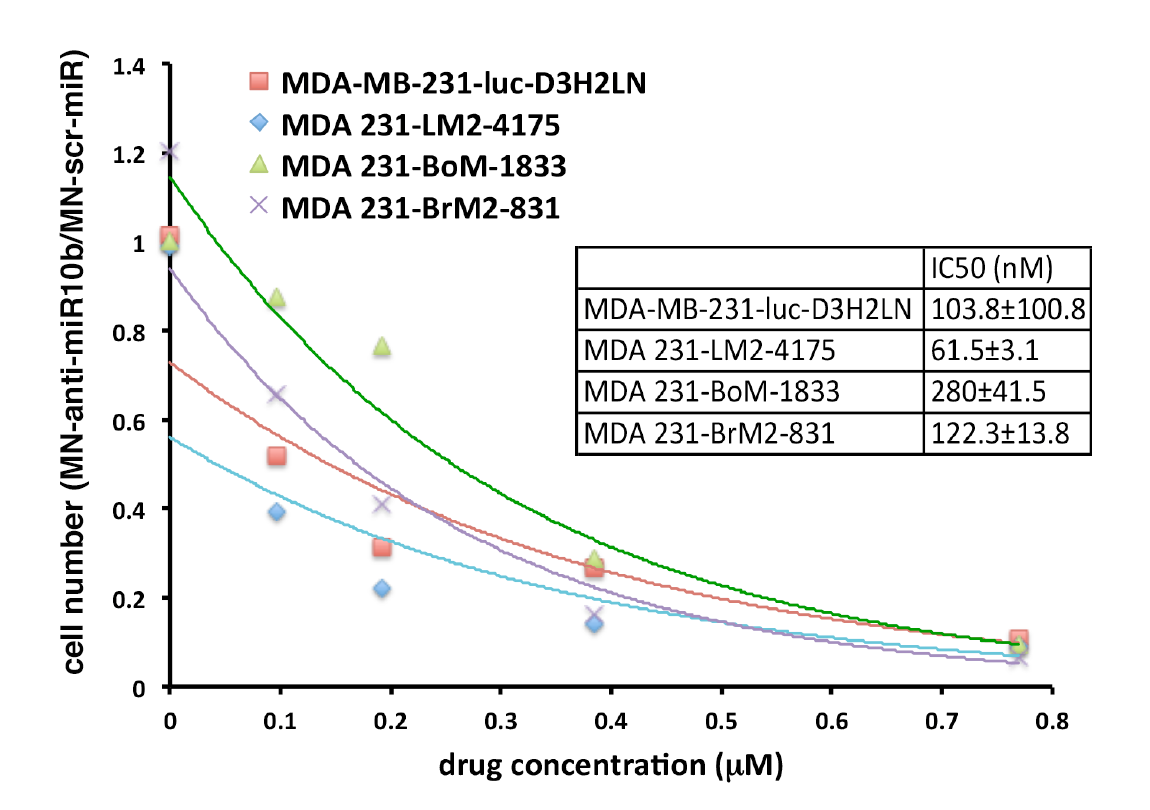


**Supplemental Figure 8.** MN-anti-miR10b effect on the viability of MDA-MB-231 cell lines derived from lymph node (D3H2LN), lung (LM2), bone (BoM), and brain (BrM2) metastases. MN-anti-miR10b inhibited the viability of all cell lines with nanomolar IC50 values.
